# Biomaterials for Treatment of Baldness

**DOI:** 10.7759/cureus.31187

**Published:** 2022-11-07

**Authors:** Palash Sahu, Harshal Ramteke

**Affiliations:** 1 Department of Dermatology, Jawaharlal Nehru Medical College, Datta Meghe Institute of Medical Sciences, Wardha, IND; 2 Department of Surgery, Jawaharlal Nehru Medical College, Datta Meghe Institute of Medical Sciences, Wardha, IND

**Keywords:** microsphere, biocompatibility, biofibre, nonthermal plasma, zeta potential, nanoparticles

## Abstract

Androgenic alopecia affects men and women today, where many factors can be the unknown cause of it. It is essential to find a good, effective, and inexpensive treatment for it, as it is a prevalent dermatological condition. Many drugs like minoxidil (MXD) and finasteride (FNS) are commercially used for its treatment but we are still lacking in terms of a complete cure for alopecia. In this study, we came up with different treatment options for alopecia and assessed their advantages and disadvantages. Not only is the treatment part is essential, but the mode of drug delivery is equally important when it comes to hair and its growth for the maximum output. The following discussion considers the idea of nanoparticles (NPs) loaded with drugs and biomaterials used for the treatment of alopecia.

## Introduction and background

Alopecia is a symptom of abnormal hair loss brought on by various factors such as heredity, hormonal imbalance, strain, and ageing. It has turned into an widespread condition that affects many humans worldwide. In order to rehabilitate alopecia patients by stimulating hair regrowth at follicular level, numerous medicinal trials have been carried out. Among these, the most popular way to treat alopecia is with medicines and the most common medicines used are minoxidil (MXD) and finasteride (FNS) [1].

Alopecia is mostly androgen dependent as higher levels of androgens in the body can shrink hair follicles and shorten the scalp hair cycle, causing hair to thin, become more brittle, and fall out more quickly; primarily, frontal hair in the temporal region is involved [2]. Changes in follicular cell protein production have a role in several of these follicular diseases. The cause of these modifications lies in the endogenous hormone testosterone, produced by the adrenal gland, a derivative of which (5-dihydrotestosterone (DHT)) alters the binding of proteins in follicular cells, causes follicular diseases, and ultimately results in alopecia [3].

**Figure 1:**
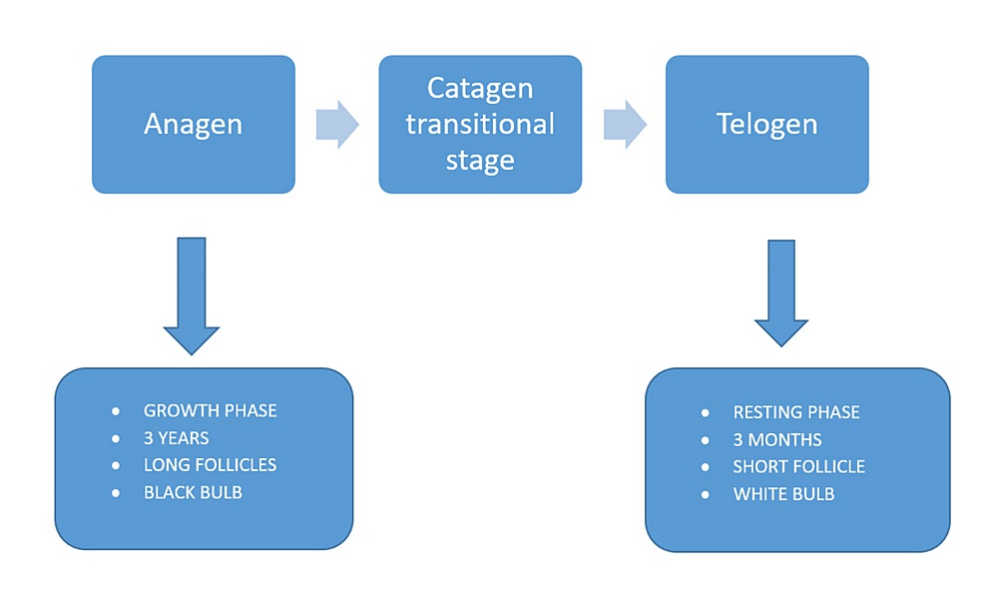
The Hair Growth Cycle Figure created by the author

Figure 1 depicts scalp hair cycle, which consists of three stages.

In the scalp hair cycle, 84% of hair are in the anagen stage, 14% are in the telogen stage, and the rest in a transitional catagen stage. Anagen pushes out the telogen, and loss of telogen occurs; more than 100 telogen loss per day is abnormal and called telogen effluvium. The loss of one anagen per day is considered abnormal as well and is called as anagen effluvium, which is commonly seen in chemotherapy patients [4]. Drugs given as a therapy for alopecia, such as FNS and MXD, cause adverse effects such as erectile dysfunction and impotence, and affects reproductive functions. Additionally, on oral administration, due to the low permeability of these drugs because of keratin layer, only a minimal amount of drug can reach the acting site. Thus, there is a need to find another route of administration. Combining these drugs with nanoparticles (NPs) so that it can penetrate easily and remain in the acting site for a longer duration of time, FNS, a poor water-soluble drug, prevents the conversion of testosterone to DHT in the periphery and is used in order to treat alopecia. This conversion results in a considerable drop in DHT levels [5,6].

Baldness is not just a medical problem but is also a cosmetic as well as a psychosocial problem. A novel method for its cure is to implant hair. One such method to transplant is the automatic biofiber hair implant method. This approach guarantees an immediate aesthetic outcome and enough hair in a short amount of time [7].

**Figure 2:**
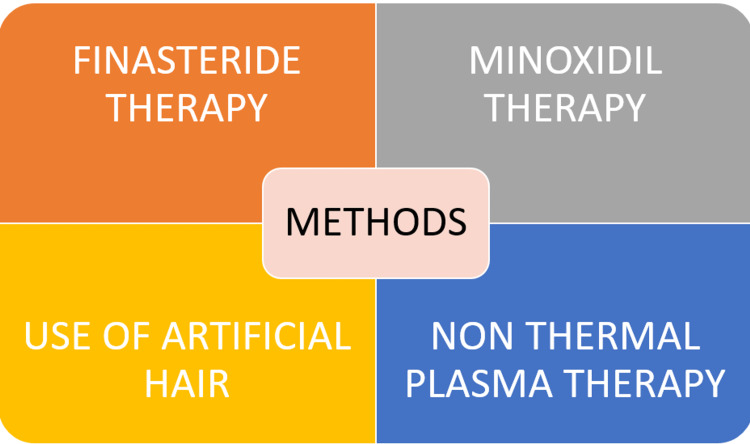
Therapies for alopecia Author's own creation

 Various methods and procedures for the different types of alopecia are shown in Figure 2.

## Review

FNS therapy

FNS, a poorly water soluble drug, prevents the conversion of testosterone to DHT in the periphery is used in order to treat alopecia. This conversion results in a considerable drop in DHT levels. FNS has poor water solubility, and thus does not have a long-lasting effect, but it can act longer if its tablets are converted to NPs, and thus the release of FNS can be prolonged by three hours [3]. The dermis and hair follicles may be accessible to polymeric NPs, which are generated using a modified emulsification/solvent diffusion method because of their mean particle size of roughly 300 nm and negative zeta potential values [2]. Zeta potential is the differential between a solid particle's surface and the bulk of a conducting liquid; the solute is more soluble in solution when its zeta potential is lower. Oral administration of FNS effectively treated several dermatological and follicular problems, especially androgenetic alopecia [8]. Advantages of FNS NPs are decreasing the drug dose, achieving solubilization of hydrophobic drugs, and eliminating side effects like erectile dysfunction, impotence, and reduced reproductive function, which are common side effects of oral FNS [2]. FNS-loaded poly D,L-lactic-co-glycolic acid (PLGA) NPs were created and characterized by modifying spontaneous emulsification with solvent diffusion. Due to its biodegradability, biocompatibility, and sustained drug release, PLGA is one of the most widely used polymers [9,10].

Previous research has demonstrated that lipids in the topmost layers of the skin often interact with negatively charged carriers like nanostructured lipid carriers (NLC) or solid lipid NPs (SLN) to provide an occlusive action. Medication retentiveness in the skin may be increased by those negatively charged NLCs and SLNs, resulting in an entire film developing on the skin surface. In skin appendages like hair follicles, other NPs like polymeric NPs may build up and serve as a long-term reservoir [2,11]. The high FNS encapsulation effectiveness in PLGA NPs indicated a good chance that PLGA and FNS would interact. Numerous researchers have established that lipophilic drugs like FNS have a high affinity for PLGA, which results in excellent encapsulation effectiveness and significant interest in the pharmaceutical business [12]. The overall conclusion is that PLGA is a valuable and secure polymer for the future application of nanotechnology in transdermal delivery systems for drug administration to the skin's surface [2]. Another method for delivering FNS is to load it with microspheres produced by a method based on the micro-electromechanical system. An experimental mouse model was created for androgenic-induced alopecia, and FNS-loaded microparticles comprising PLGA and FNS at a 4:1 ratio were employed. Post 10 weeks, the positive control group that received FNS orally experienced a pilosity of 86.7% (overall hair growth of 60%, partial hair growth of 26.7%).

In comparison, the group that received FNS-loaded microspheres experienced hair growth of 93.3% (total hair growth of 60%, partial hair growth of 33.3%) [13]. FNS-loaded polycarbonate and poly propylene carbonate maleate microparticles have demonstrated regular drug release in vitro for up to five-to-six weeks. Additionally, topical administration of polystyrene-b-poly acrylic acid polymersomes coated with chitosan enhances the drug’s ability to absorb and aggregate in the skin layer [14].

MXD therapy 

In PLGA-grafted hyaluronate NPs made using the W/O/W solvent evaporation technique, MXD and rhodamine B (Rho B) are enclosed (HA-PLGA/MXD NPs, HA-PLGA/Rho B NPs) and are found to be an effective method for the treatment of alopecia. HA-PLGA/MXD NPs and HA-PLGA/Rho B NPs have been produced effectively. Additionally, it has been shown that HA-PLGA NPs may sufficiently enter cells without causing a lot of cell cytotoxicity [15]. Regular MXD administration has the drawbacks of limited transdermal transport efficiency and several adverse effects, including inflammation, tolerance, and low blood pressure [16]. Due to this, many researches has been done to improve the transdermal drug delivery effectiveness of MXD administration to the skin as well as the therapeutic productiveness in alopecia, employing transdermal pathways such as sweat glands, hair follicles, or microneedle arrays [15-17].

It is well recognized that MXD has a vasodilatory action and hastens the release of factors for hair regrowth. With HA-PLGA/MXD NPs, Jeong et al. have created a transdermal drug delivery system for the therapy of alopecia [15]. For many biological uses, HA is a well-known common polysaccharide [15]. It is known that PLGA is permeable to the skin because of its hydrophobicity, and several earlier studies have described transdermal distribution utilizing PLGA [15]. The best alternative to injection with a needle is transdermal medication administration. However, there are certain drawbacks, including poor bioavailability and delivery efficiency. MXD is frequently used in the treatment of alopecia, although there are drawbacks to its usage when it is administered to the skin. We created unique transdermal carriers in the shape of NPs to boost the transport effectiveness of MXD [18,19], The effectiveness of MXD varies from person to person, and when treatment is stopped, its hair-growth impact wears off and hair loss gradually returns. Furthermore, aftereffects of MXD, such as itchiness, dandruff, dehydration, and allergic contact dermatitis, are possible [20].

We employed an emulsification approach using solvent evaporation to utilize each material with good qualities in delivering MXD efficiently. The emulsion approach has reduced particle size as a drug delivery carrier, improved hydrophobic drug stability, and more accessible storage. In order to do this, we used the water-in-oil-in-water emulsion approach, which has been extensively researched in drug administration systems [21,22]. Through the penetration test, we could confirm that HA-PLGA NPs finally passed through hair follicles. Eight hours later, the fluorescence intensity in the epidermis and follicular cells was almost consistent [15]. However, the intensity in the dermis and follicle cells rose while the intensity in the stratum corneum generally continued to decline. Thus, according to these data, HA-PLGA NPs delivered to follicle cells were dispersed to the dermis and required eight hours to reach the surrounding tissue. Combining MXD with HA-conjugated NPs may benefit the treatment of baldness [15].

Artificial hair as therapy

Initially, the use of artificial hair as a therapy was not so common as there were high chances of rejection of implanted hair, severe infection, inflammation in scalp, and breakage of implanted hair in the scalp, among others, due to lack of proper techniques and suitable materials. However, recent advances in medical sciences have led to a suitable biocompatible fiber called biofibers [7]. In collaboration with university departments, European biomedical businesses started investigating synthetic hair. Biocompatible fibers (Biofibre®) were created in 1993 by Medicap in Italy. Clinical trials and histology investigations started in 1993 with good outcomes, which prompted more investigation into biocompatible materials and the application of medical protocols [23,24]. In 2013, Medicap Italy introduced the first device for Biofibre® hair implants, which has significant advantages for the procedure [7]. The latest high-density variation of Biofibre®, known as MHD® hair, was developed in 2014 and enabled three times as many implants to produce the same amount of hair. For the anterior hairline, Biofibre® single in number is advised to make sure a more natural aesthetic outcome. Currently, such fibers are exclusively utilized for the crown region because they only require light maintenance and produce results quickly. The primary requirements are biocompatibility, traction resistance, lack of capillarity, physical-chemical stress resistance, decreased tissue damage, and aesthetically pleasing attributes. The biocompatibility and safety standards for medical devices set out by international standards bodies are fully met by Biofibre® medical hair prosthesis fibers [25]. The tools that are employed throughout the surgery are equally crucial. The hooked needles are less painful when administering a drug, depending on their diameter and form. Additionally, these tools guarantee that the patient will be as comfortable as possible while the doctor works. The automatic Biofibre® hair implant system offers a helpful response to such a need [7].

The procedure for Biofibre® hair implant requires a minor surgery, Both male and female patients can take it for diffuse alopecia or hair thinning. Without needing a donor location, this approach guarantees an immediate aesthetic outcome and enough hair in a short time. It is a reasonably gentle procedure that does not require hospitalization. To improve final cosmetic results or in situations when the donor region is not in excellent condition, it may be used alone or in conjunction with other hair restoration techniques. Additionally, it is carried out to treat scars or scalp burns [26,27]. There are certain disadvantages of this method. Placement sites like the temples, low anterior hairline, scalp regions, pathologically atrophic scalp, along with fragile dermal group of cells such as sideburns, are not recommended for this procedure. This therapy is not recommended for those with diabetes mellitus (DM), hepatitis A, B, or C, autoimmune disorders, long-standing scalp conditions, severe psychological disorders, unstabilized alopecia areata, poor personal cleanliness, or jobs requiring work in filthy or dusty conditions either [28]. Before the implant, the patient must abstain from smoking, consuming alcohol, and consuming salicylic acid for a minimum of three days. The region where the implant will be placed has to be thoroughly cleaned before beginning. Another drawback of this method is that the implanted hair will not grow. It has to be periodically touched up to maintain the desired aesthetic outcome [7,29].

Non-thermal plasma (NTP) therapy in alopecia

NTP is frequently used to clean and personalize biomaterials. Nevertheless, the effects of NTP therapy on hair growth roots and related processes are yet unknown. It can, however, heal and improve skin function. In this article, we suggest an air-based NTP therapy that produces extrinsic nitric oxide (eNO) as a preventative measure for the evident loss of hair [30]. When air-based NTP is applied topically, significant levels of eNO may be immediately measured using a small-sized electrode NO sensing system in the mouse's outer skin, eNO produced by NTP also stimulates hair development by enhancing the formation of capillary tubes and increases cell division, production of hair, and formation of newer blood vessels [31]. Improvement in hair growth results from NTP therapy, which increases the stem cell activity of the hair follicle to support the anagen:catagen:telogen ratio. NTP is an effective advancement and a potential treatment strategy for hair regeneration. One of the four basic states of matter is plasma, which has been extensively employed to clean and alter the surface of biomaterials. According to reports, NTP has the potential to be used clinically in the treatment of skin pathologies such as pressure ulcers, acne, and chronic wounds [30-32]. 

Additionally, transforming growth factor (TGF), vascular endothelial growth factor (VEGF), granulocyte macrophage colony stimulating factor (GM-CSF), and epidermal growth factor (EGF) levels in mouse skin are significantly elevated by NTP, which favors hair growth [33]. Reactive oxygen and nitrogen species, such as H_2_O_2_, superoxide, hydroxyl free radicals, and NO, are also produced by NTP. These reactive oxygen species (ROS) generally have potent oxidative characteristics that can change biological components such as lipids, proteins, and DNA/RNA [34]. However, NO may also operate as a regulator of activity at cellular level, a signal molecule for cellular processes, and a catalyst for immunological deficiency, raised cell division and stimulation of cells, which results in neovascular genesis, cell engulfment by phagocytes, and collagen formation [31-35]. NTP is a simple, non-intrusive technique that may even be created as a wearable item, like a reusable pad of 32 inches. The development of NTP technology and its use might significantly impact how well specific skin problems, such as hair loss, are controlled. We initially created a dielectric barrier discharge-NTP system and assessed its efficacy before using NTP in the in vitro and in vivo hair loss models. The produced plasma was highly efficient during the 2.30-2.34 kV range. A professional organization, Korea Testing Certification (KTC), conducted additional testing on the NTP device's electrical and electromagnetic safety. It was determined that the NTP advanced device was not hazardous for usage in skin science advancement purposes [30].

Table 1 summarizes the key points about the advantages and disadvantages of drugs/biomaterials used to treat baldness.

**Table 1:** Advantages and disadvantages of drugs/biomaterials used for the treatment of alopecia FNS: Finasteride; PLGA: Poly D,L-lactic-co-glycolic acid; NP: Nanoparticle; HA-PLGA: Hyaluronic acid poly D,L-lactic-co-glycolic acid; MXD: Minoxidil; DM: Diabetes mellitus; NTP: Non-thermal plasma

DRUG/BIOMATERIAL USED	ADVANTAGE	DISADVANTAGE
FNS-loaded PLGA NPs	Can penetrate easily and remain in the acting site for a longer duration of around 5-6 weeks; release of the drug can be prolonged by 3 hours	Erectile dysfunction, impotence, ejaculation disorders
MXD encapsulated with HA-PLGA	Less cytotoxic to cells compared to other drugs used for hair fall; good skin permeability; increased hair thickness	Regular use of MXD can cause inflammation of the scalp, tolerance to the drug, hypotension due to its vasodilatory action, dandruff and, itchiness. As treatment stops, its hair growth impact wears off
Use of artificial hair (Biofibre® hair implant)	Immediate aesthetic outcome; enough hair in a short duration of time; can be used to treat scars or scalp burns; no need for any donor as Biofibres® are artificially manufactured	The implanted hair will not grow; it is a surgical procedure so patients may resist this; pathologically atrophic scalps are not recommended placement sites for this procedure; this procedure is not advised in patients with DM, Hep A, B, C, and autoimmune disorders; chances of severe infections.
NTP therapy	Can also be used in skin pathologies such as pressure ulcers, acne, etc.	Under trial

## Conclusions

The effectiveness of superficial regimens for the management of alopecia depends critically on the medication's ability to penetrate the skin sufficiently to have the desired therapeutic effect. Currently, the most successful way to treat alopecia is using MXD-encapsulated HA-PLGA NPs. PLGA is employed as a polymer for FNS distribution to hair follicles and is shown to be a safer and more beneficial polymer with a long-lasting impact of up to five-to-six weeks. MXD was efficiently absorbed by cells at sufficient concentrations without irreversible cell damage and toxicity. These outcomes suggest that HA-PLGA NPs may be utilized to develop a successful and efficient platform for treating various illnesses and are suitable for the transdermal delivery of MXD for treating alopecia. But it is also observed that these drugs have some side effects like impotence, ejaculation disorder, hypotension, etc. As these side effects are really subjective and mainly depend on the patient’s reaction to the drug, the final choice of whether to go with drugs or with the use of an artificial hair implant has to be made by the patient. In cases of androgenic alopecia, hair loss, and scarring, artificial hair transplantation using a biocompatible fiber may be an option for individuals who still choose not to receive pharmacological therapy. It may be a successful hair restoration procedure for male and female patients. NTP therapy is another alternative for treatment. It is a straightforward, non-invasive technique that may be implemented as a reusable product, such as an attachable pad of 32 inches. Due to their small size, ease of production, and simplicity of use, NTP devices also have the advantage of being portable.
